# Generating EQ-5D-3L health utility scores from the Edinburgh Postnatal Depression Scale: a perinatal mapping study

**DOI:** 10.1007/s10198-023-01589-4

**Published:** 2023-04-24

**Authors:** Elizabeth M. Camacho, Gemma E. Shields, Carolyn A. Chew-Graham, Emily Eisner, Simon Gilbody, Elizabeth Littlewood, Dean McMillan, Kylie Watson, Pasco Fearon, Deborah J. Sharp

**Affiliations:** 1 School of Health Sciences, University of Manchester, Jean McFarlane Building, Oxford Road, Manchester, M13 9PT UK; 2 School of Medicine, Keele University, Keele, UK; 3 Greater Manchester Mental Health NHS Foundation Trust, Manchester, UK; 4 Hull York Medical School and Department of Health Sciences, University of York, York, UK; 5 Department of Health Sciences, University of York, York, UK; 6 Manchester University NHS Foundation Trust, Manchester, UK; 7 Research Department of Clinical, Educational and Health Psychology, University College London, London, UK; 8 Centre for Academic Primary Care, University of Bristol, Bristol, UK

**Keywords:** Utility, Mapping, EQ-5D, Edinburgh Postnatal Depression Scale, Perinatal depression

## Abstract

**Background:**

Perinatal depression (PND) describes depression experienced by parents during pregnancy or in the first year after a baby is born. The EQ-5D instrument (a generic measure of health status) is not often collected in perinatal research, however disease-specific measures, such as the Edinburgh Postnatal Depression Scale (EPDS) are widely used. Mapping can be used to estimate generic health utility index values from disease-specific measures like the EPDS.

**Objective:**

To develop a mapping algorithm to estimate EQ-5D utility index values from the EPDS.

**Methods:**

Patient-level data from the BaBY PaNDA study (English observational cohort study) provided 1068 observations with paired EPDS and EQ-5D (3-level version; EQ-5D-3L) responses. We compared the performance of six alternative regression model types, each with four specifications of covariates (EPDS score and age: base, squared, and cubed). Model performance (ability to predict utility values) was assessed by ranking mean error, mean absolute error, and root mean square error. Algorithm performance in 3 external datasets was also evaluated.

**Results:**

There was moderate correlation between EPDS score and utility values (coefficient:  – 0.42). The best performing model type was a two-part model, followed by ordinary least squared. Inclusion of squared and cubed covariates improved model performance. Based on graphs of observed and predicted utility values, the algorithm performed better when utility was above 0.6.

**Conclusions:**

This direct mapping algorithm allows the estimation of health utility values from EPDS scores. The algorithm has good external validity but is likely to perform better in samples with higher health status.

**Supplementary Information:**

The online version contains supplementary material available at 10.1007/s10198-023-01589-4.

## Introduction

Depression during pregnancy or in the first year after having a baby (perinatal depression; PND) is experienced by up to 17% of mothers globally [1]. The National Health Service in England reports that up to 27% of mothers experience mental health problems in the perinatal period [2]. Based on the annual number of births, this equates to more than 160,000 women per year in England and Wales [3]. The lifetime societal burden of PND and other perinatal mental health conditions is substantial, estimated at £8.1bn for each one-year cohort of births [4]. This includes £1bn of NHS services, in addition to costs associated with time off work, marriage breakdown, and social services support.

As with depression experienced outside of the perinatal period, the symptoms of PND include: low mood/energy, loss of interest in pleasurable activities, marked tiredness, disturbed sleep, reduced appetite, low self-esteem/self-confidence, and feelings of guilt or worthlessness [5]. Around 50% of women experience low mood in the first few weeks following birth this is distinct from PND, and often mild and transient [6]. Established risk factors for PND include a history of prior depression or anxiety, poor partner relationship, lack of social support, stressful life events during pregnancy, low socio-economic status, unintended pregnancy, and domestic violence [7, 8]. PND can have important implications for the life-course of mothers and children; depression during pregnancy is strongly associated with both depression and anxiety following childbirth [4]. Other important long-term impacts include developmental delays and behavioural problems for children, and family instability [2, 9]. PND is often comorbid with perinatal anxiety with as many as two-thirds of women with PND also having an anxiety disorder [10, 11].

Psychological therapy and/or antidepressant medication are effective at treating PND for many women [12, 13]. When PND is untreated it is associated with long-term and/or recurrent mental ill health in up to 70% of women [14]. Although less is known about which treatments are cost-effective [15]. A systematic literature review found a limited amount of published cost-effectiveness evidence in relation to PND, and it was not possible to draw conclusions to guide decision-makers [15].

In England, the National Institute for Health and Care Excellence (NICE) make decisions on healthcare commissioning, namely recommending whether healthcare interventions should be provided by the health service. These decisions are based on a range of factors including clinical effectiveness and cost-effectiveness evidence. NICE recommend that the measure of health benefit used in cost-effectiveness analysis is the quality-adjusted life year (QALY) which is calculated using health utility values derived from the EQ-5D [16].

The EQ-5D is a generic measure of health (i.e. can be used in many different health conditions) and captures health over 5 domains [17]. The five domains incorporate both physical and mental aspects of health and are: mobility, self-care, usual activities, pain/discomfort and anxiety/depression. The EQ-5D is the most widely-used instrument for measuring health in cost-effectiveness analysis [18]. Typically, in research studies treatment effects are captured using disease-specific measures rather than generic instruments like the EQ-5D. For example, the Edinburgh Postnatal Depression Scale (EPDS) is a validated tool for measuring the symptoms of PND [19]. It is used in some routine care settings, and is the most commonly used tool in research settings [20].

NICE recommend that where primary EQ-5D data are not available, utility values can be estimated from disease-specific measures. Disease-specific measures can be used to predict health utility values by using an algorithm that maps between the two [21]. The benefit of doing this is that studies which did not collect EQ-5D data can be revisited and the health benefit recalculated in terms of health utility and QALYs. This makes the findings more directly accessible and relevant to decision-makers like NICE, and hence more likely to shape health policy and practice. This is greatly needed in relation to interventions for PND where there is currently a lack of cost-effectiveness evidence [15].

Mapping between disease-specific and generic health measures is a well-established methodological approach. There is a curated database of published mapping studies which currently reports over 180 examples [22]. According to a review of published mapping studies this has not been done before between the EQ-5D and the EPDS [23].

The aim of the analysis reported in this paper, was to develop and validate a mapping algorithm between the EPDS and 3-level version of the EQ-5D (EQ-5D-3L).

## Methods

This analysis has been conducted according to current good practice guidance for mapping studies [21, 24]. As per the guidance there are three key stages to mapping: establishing an estimation dataset, developing the algorithm, and validating the algorithm. Our approach to each stage is outlined in more detail below. The aim was to produce a mapping algorithm between the Edinburgh Postnatal Depression Scale (EPDS) and the EQ-5D-3L, to aid future research, in particular cost-utility studies.

### Data source

BaBY PaNDA was an observational longitudinal cohort study of pregnant women in Yorkshire, England, who were followed until one year postpartum [7]. The full protocol for the BaBY PaNDA study has been published previously [25]. In brief, 391 pregnant women were recruited to the BaBY PaNDA study between July 2013 and August 2014. Ethical approval for the data collection was granted by North East – York Research Ethics Committee (REC) on 23 April 2013 (reference number: 11/NE/0022) and was subsequently approved by the relevant NHS trust’s research and development (R&D) committees. All participants provided written consent to participate in the study. Outcome measures were collected when participants were at around 20 weeks gestation and 3 months and 1 year postnatally. Participants were asked to complete the EQ-5D-3L and EPDS at each assessment. Both measures were completed by participants on their own behalf. Any assessment where participants completed both measures were included in the analysis.

### Outcome measures

The EQ-5D-3L was developed between 1987 and 1991 by an inter-disciplinary five-country group of researchers with the aim of measuring and valuing health as part of cost-effectiveness analysis [26]. The EQ-5D-3L asks respondents rate their health over the five domains (mobility, self-care, usual activities, pain/discomfort, and depression/anxiety) on a scale of 1 to 3, with 1 indicating no problems on that domain, 2 denoting some problems, and 3 denoting extreme problems. Respondents are asked to consider their health on the day they complete the measure. Each profile of responses across the 5 domains can be matched to a corresponding health utility value. Someone who reports ‘no problems’ on all health domains (i.e. health profile 11111) is said to have full health and is assigned a utility value of 1. For each domain with less than perfect health there is an associated utility decrement. A utility value of 0 is equivalent to being dead and values less than 0 represent health states ‘worse than death’ e.g. extreme problems on all health domains. In the UK there is an accepted set of utility values (or a 'tariff') that is assigned to each health profile [27]. The UK tariff was elicited from the health preferences of a sample of people from the general public in the UK. This published UK tariff of utility values was used in the present study [27]. In this tariff, utility values range from 1 to  – 0.594.

The Edinburgh Postnatal Depression Scale (EPDS) was developed and validated by Psychiatrist John Cox over 30 years ago as a self-completion questionnaire to help improve the identification of PND [19, 28]. The EPDS was not designed to be a diagnostic instrument but rather to be used for PND case-finding. The EPDS asks respondents to answer 10 questions about how they have felt over the previous 7 days. Each question is scored between 0 and 3, with total scores ranging between 0 and 30. A higher score indicates more/greater symptoms of depression. Total EPDS scores are compared against a cut-off score to identify cases of PND. There is a balance to be struck between sensitivity (ability to identify true positive cases of PND) and specificity (ability to identify true negative PND). A range of cut-off scores are used in practice and in different contexts, but scores commonly used to denote caseness for postnatal depression are scores ≥ 10 or ≥ 13 [19, 29, 30].

Summary statistics were produced for both measures and the distribution of EQ-5D responses and utility values observed in the sample were explored graphically.

### Conceptual overlap

The degree of conceptual overlap between the EQ-5D-3L and the EPDS was explored using Spearman rank correlations. Correlation coefficients were generated between total EPDS score and utility index value (to determine if direct mapping is appropriate), and also between total EPDS score and each domain of the EQ-5D-3L (to determine if response mapping is appropriate). Spearman rank correlation was used to explore the individual EQ-5D-3L domains as the responses on the domains (1 = no problems, 2 = some problems, 3 = extreme problems) are ranks. The same approach was also used for utility index values as they were not normally distributed and Spearman rank correlation does not assume that data are normally distributed. Correlations were assessed according to the following categorisation: very weak (0–0.19), weak (0.20–0.39), moderate (0.40–0.59), strong (0.60–0.79), and very strong (0.80–1). A correlation that was least moderately strong was considered appropriate to proceed with mapping. P-values for the correlation coefficients were also reported.

### Model development

The correlation coefficients indicated that only direct mapping (i.e. between total EPDS score and utility index value) was appropriate. Six alternative regression model types were explored: ordinary least squares (OLS), Tobit, generalised linear model (GLM), two-part, adjusted limited dependent variable mixed model with two latent classes (ALDVMM-2), and adjusted limited dependent variable mixed model with three latent classes (ALDVMM-3). OLS, Tobit, GLM, and two-part models are the most frequently used model types in studies mapping to the EQ-5D [31]. OLS is easy to interpret and the top most common estimation methods used [31, 32], however it only allows for a simple linear relationship between the measures. The Tobit model was used because it can be specified to restrict predicted utility values to the range of possible values that can occur according to the UK tariff (i.e.  – 0.549 to 1). The GLM was specified to use a Gaussian family and log-link function as this allows for the skewed distribution of utility values and censors the predicted utility values to the upper bound of 1. The two-part model was used to take into account clustering of utility values at 1 (i.e. full health). The first part of the model used a logistic regression to predict whether someone is in full health. The second part of the model used an OLS model to predict the utility values of the people with less than full health. Both the GLM and two-part models predict disutility (i.e. decrement from full health) which is then transformed back into utility. The ALDVMMs make two key allowances for the features of utility index values [33]. The adjusted limited dependent variable incorporates the upper bound at full health and also the gap between full health and the next highest possible utility value (0.883). The mixed model framework accounts for the typically bi- or tri-modal distribution observed in utility index values. Two and three latent classes were explored as per the methods used by the developers of the approach [33].

For each model type, 4 model specifications were estimated: (1) total EPDS; (2) total EPDS, age; (3) total EPDS, age, total EPDS^2^, age^2^; (4) total EPDS, age, total EPDS^2^, age^2^, total EPDS^3^, age^3^. The model specifications with higher order polynomials are henceforth referred to as “squared covariates” and “cubed covariates” respectively. Additional covariates were not included in the model so that the mapping algorithm could be used in a wide range of datasets with only these basic variables available, rather than restricting its use to large, complex, datasets.

### Model performance

Model performance was explored by comparing the mean, SD, minimum, and maximum of predicted utility values to the observed data. The predictive ability of the different model types and specifications within the estimation sample was assessed by comparing the utility values they predicted to their respective observed values. This involved calculating mean error (ME), mean absolute error (MAE), and root mean squared error (RMSE) for each model. For each metric (ME, MAE, RMSE) the models were ranked (lower rank indicating better performance) and each of the 3 ranks were added together to generate an overall rank. The model with the smallest value for overall rank was considered to be the best-performing model. The utility values predicted by the best-performing model were plotted again observed utility values as a visual representation of model fit.

The predicted utility values from the best-performing model and the simplest model (OLS model with no additional covariates) were explored at specific EPDS scores and thresholds commonly used to denote caseness for postnatal depression (10 and 13 [19, 29, 30]).

### External validation

The predictive ability of specifications 1 (no additional covariates) and 4 (cubed covariates) for the best two performing model types were explored in the three external datasets. In all three datasets, any assessment point where participants completed both measures were included in the validation. One dataset was from the SHIP study, a randomised trial of self-hypnosis for intrapartum pain management in nulliparous women [34] with 1059 pairs of observed data on the EPDS and EQ-5D-3L. The second dataset was from Minding The Baby, a randomised trial of a parenting intervention for young mothers, with 319 pairs of data on the EPDS and EQ-5D-3L [35]. Participant-level data on age were not available for secondary analysis therefore an age of 19 years was assumed for all participants as the study recruited mothers aged 19 or under, or those aged 20–25 with high social deprivation (defined as: (1) eligible for means-tested benefits;( 2) not entitled to employer maternity pay; or (3) living in a postcode falling within the highest quintile of social deprivation or sheltered accommodation). The third dataset was from a study of women with a confirmed diagnosis of postnatal depression, the RESPOND study [36]. This was a randomised trial of antidepressants versus a psychosocial intervention, with 245 pairs of data on the EPDS and EQ-5D-3L. Performance of the models in the external datasets was measured in the same way as internal performance, in terms of ME, MAE, and RMSE ranks. Graphs of observed and expected utility values were plotted for the best-performing model, and of errors/absolute errors against total EPDS score within each data sample.

## Results

### Descriptive statistics

Within the BaBY PaNDA dataset there were 1068 observations with complete data on the EQ-5D-3L and EPDS, from 390 participants. Participant characteristics and summary statistics are reported in Table 1. The mean (SD) age of the sample was 31.2 (5.1). The mean (SD) EPDS score was 5.5 (4.6) and observed scores ranged from 0 to 22 (out of a possible 30).

**Table 1:** Sample characteristics of BaBY PaNDA participants

n = 1068 observations	Mean (SD)	Range (min, max)
Age at baseline (years)	31.2 (5.1)	16, 46
EPDS score (0–30)	5.5 (4.6)	0, 22
EQ-5D-3L utility index value	0.890 (0.150)	– 0.429, 1
	n	(%)
Full health (EQ-5D-3L profile 11111)	476	(44.6)
Negative utility index value	2	(0.2)

EPDS = Edinburgh Postnatal Depression Scale

**Fig. 1:**
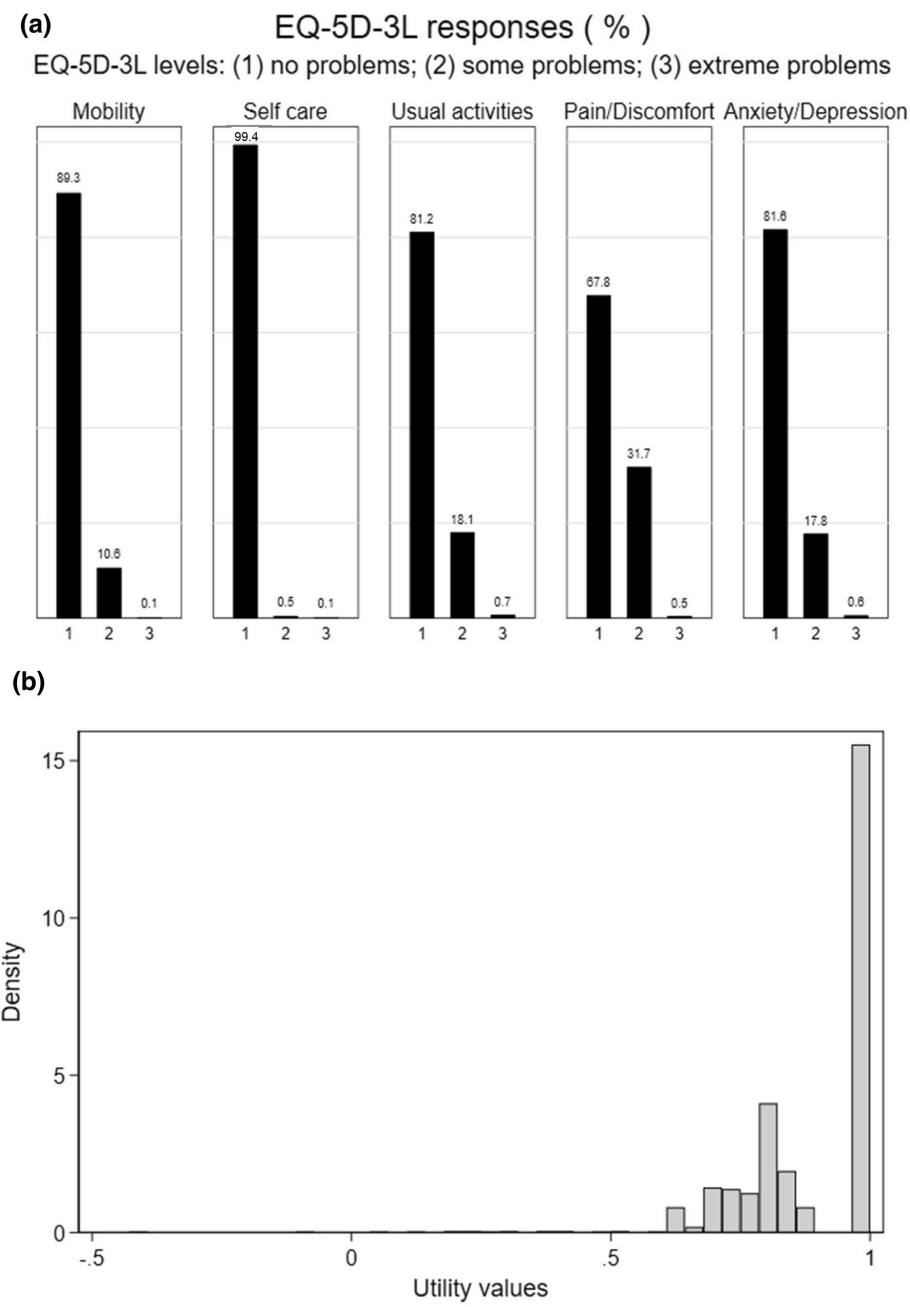
Proportions of response to items in the EQ-5D-3L (**a**) and distribution of EQ-5D-3L utility index values (**b**) (total sample, n = 1068)

The proportion of observations with the different responses on the EQ-5D-3L are shown in Fig. 1 (panel a). Across all five health domains, the majority of the observations had no problems (67.8–99.4%). A very small proportion had extreme problems on any domain (0.1–0.7%).

The mean (SD) EQ-5D-3L utility index value was 0.890 (0.150). Figure 1 (panel b) shows the distribution of utility index values in the BaBY PaNDA sample. The data are highly skewed and there is a clustering of observations at a utility value of 1 (full health) which accounts for 44.6% (476/1068) of observations. There was a secondary peak at 0.796, with 12.8% (137/1068) of the observations having this value. This is the utility value for the EQ-5D-3L profile 11121, which corresponds to no health problems in all domains apart from pain/discomfort where some problems are reported. Two observations (0.2%) had negative values (i.e. health status ‘worse than death’).

The correlation coefficients between total scores and domains of the EQ-5D-3L and the EPDS are shown in Table 2. There was a moderate negative correlation between total EPDS score and utility index value, as would be expected as higher utility values and lower EPDS scores indicate better health. There was a moderate positive correlation between the anxiety/depression domain and EPDS score, but weak positive correlations between the other domains and EPDS score. These correlations were positive as higher scores indicate worse health on both measures. Due to the majority of the correlations between EPDS score and the EQ-5D-3L domains being weak, response mapping was not conducted, and the remainder of the analyses focused on direct mapping only (i.e. between EPDS total score and EQ-5D-3L utility value).

**Table 2:** Spearman rank correlation coefficients between EQ-5D-3L (total score and 5 domains) and EPDS total score

EQ-5D-3L	EPDS total score	
	Coefficient	p-value
Utility index value	– 0.42	< 0.0001
Mobility	0.21	< 0.0001
Self-care	0.05	0.14
Usual activities	0.28	< 0.0001
Pain	0.22	< 0.0001
Anxiety/depression	0.55	< 0.0001

Note strength of correlation in absolute terms: very weak (0–0.19), weak (0.20–0.39), moderate (0.40–0.59), strong (0.60–0.79), and very strong (0.80–1)

Table 3 presents information on the performance and predictive ability of the regression models for all 4 model specifications ([1] no covariates, [2] covariates, [3] covariates squared, and [4] covariates cubed) and 6 model types (linear OLS, Tobit, GLM, two-part, ALDVMM-2 component, ALDVMM-3 component). Across all the models, the maximum predicted value is less than one (i.e., predicted values equal to one are absent). Furthermore, the minimum predicted values range from 0.347 to 0.683, which are far higher than the observed minimum value of  – 0.429. The means of the predicted values range between 0.883 and 0.896, which are not too dissimilar to the observed mean of 0.890. However, due to the smaller range of predicted values, the variation in these values (SDs between 0.063 and 0.086) is smaller than for the observed data (SD 0.150).

**Table 3:** Model performance and predictive ability for the mapping models within the BaBY PaNDA dataset (n = 1084)

MODEL	Mean	SD	Min	Max	Log likelihood	ME	MAE	RMSE	ME rank	MAE rank	RMSE rank	Overall rank
*Actual data*	*0.890*	*0.150*	* – 0.429*	*1*								
OLS1	0.890	0.068	0.648	0.971	628.84	**0.00000**	0.10253	0.13429	4	7	12	23
OLS2	0.890	0.068	0.641	0.982	629.27	**0.00000**	0.10252	0.13424	5	6	10	21
OLS3	0.890	0.068	0.601	0.967	630.56	**0.00000**	0.10332	0.13408	3	14	6	23
OLS4	0.890	0.069	0.651	0.994	632.40	**0.00000**	0.10313	0.13385	7	11	1	19
TOBIT1	0.896	0.076	0.549	0.964	– 363.35	– 0.00628	0.10196	0.13462	20	4	14	38
TOBIT2	0.896	0.076	0.545	0.966	– 363.30	– 0.00629	0.10195	0.13461	21	3	13	37
TOBIT3	0.896	0.074	0.592	0.968	– 362.65	– 0.00612	0.10119	0.13425	18	2	11	31
TOBIT4	0.896	0.075	0.644	0.976	−361.64	– 0.00612	0.10094	0.13419	19	1	9	29
GLM1	0.888	0.063	0.533	0.940	623.22	0.00204	0.10680	0.13500	12	16	17	45
GLM2	0.888	0.063	0.530	0.941	623.24	0.00206	0.10681	0.13500	13	17	16	46
GLM3	0.890	0.069	0.653	0.966	631.95	– 0.00017	0.10290	0.13390	10	9	4	23
GLM4	0.890	0.069	0.660	0.969	632.00	– 0.00007	0.10297	**0.13389**	9	10	3	22
TWOPART1	0.890	0.067	0.683	0.963	– 313.06	**0.00000**	0.10254	0.13417	8	8	8	24
TWOPART2	0.890	0.067	0.676	0.967	– 312.63	**0.00000**	0.10247	0.13409	6	5	7	18
TWOPART3	0.890	0.069	0.643	0.956	– 309.80	**0.00000**	0.10318	0.13394	2	13	5	20
TWOPART4	0.890	0.069	0.637	0.971	– 309.16	**0.00000**	0.10314	0.13387	1	12	2	15*
ALDVMM(2)1	0.883	0.086	0.351	0.925	– 166.12	0.00679	0.11075	0.13952	23	22	22	67
ALDVMM(2)2	0.883	0.086	0.347	0.932	– 164.34	0.00676	0.11083	0.13964	22	23	23	68
ALDVMM(2)3	0.885	0.071	0.641	0.932	– 157.32	0.00494	0.10688	0.13511	16	19	18	53
ALDVMM(2)4	0.885	0.071	0.583	0.939	– 156.39	0.00492	0.10682	0.13494	15	18	15	48
ALDVMM(3)1	0.885	0.081	0.403	0.925	– 155.21	0.00428	0.11023	0.13865	14	20	20	54
ALDVMM(3)2	0.885	0.084	0.377	0.932	– 152.54	0.00504	0.11067	0.13927	17	21	21	59
ALDVMM(3)3	0.888	0.072	0.650	0.936	– 131.53	0.00185	0.10627	0.13528	11	15	19	45

The assessment of model performance is based on ME, MAE, RMSE ranks, with ‘1’ indicating the closest fit to the observed data

Bold numbers indicate a mean error of 0 to at least 5 decimal places

*Best-performing model

Note that for the 3-component ALDVMM model with cubed covariates convergence was not achieved

SD = standard deviation; ME = mean error; MAE = mean absolute error; RMSE = root mean squared error

Model specifications: (1) total EPDS; (2) total EPDS, age; (3) total EPDS, age, total EPDS^2^, age^2^; (4) total EPDS, age, total EPDS^2^, age^2^, total EPDS^3^, age^3^

**Fig. 2:**
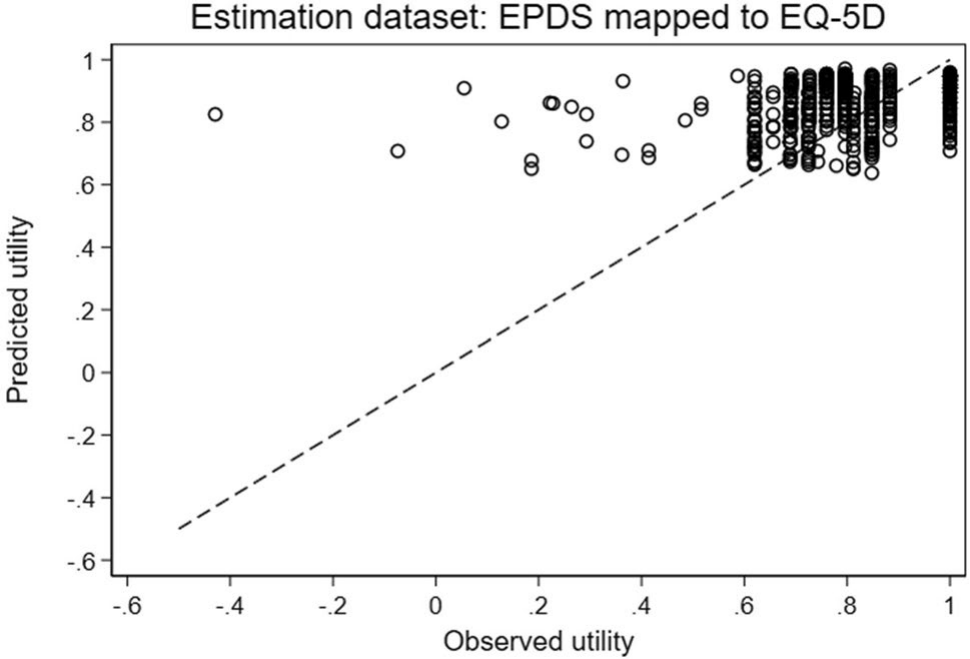
Predicted and observed EQ-5D-3L utility index values of best-performing model (two-part model with cubed covariates)

The model with the highest ranked predictive ability was the two-part model with the cubed covariates. This model had the lowest ME and second lowest RMSE (0.13387). A graph showing the observed utility index values and the values predicted by this model is shown in Fig. 2. According to the graph, the model appears to be best at predicting values between approximately 0.7 and 0.9. As expected, due to the shape of the distribution of the observed values, the model overpredicts at the low end and underpredicts at the high end of the range of utility index values. However, all of the OLS and two-part models had MEs of zero to 5 decimal places, suggesting that on average (i.e. across the whole range of values) these models neither over- nor under-predicted utility index values.

The model with the top RMSE rank was the OLS model with cubed covariates (0.13385), although this was very close to the RMSE of the top-performing model. RMSEs ranged from 0.13385 to 0.13964 which represents a percentage error of approximately 10% of the overall utility index value range. MAEs ranged from 0.10094 to 0.11083 which represents a percentage error of approximately 8% of the overall utility index value range. The 4 Tobit models were the 4 top-ranked models in terms of MAE, but were among the worst-performing models in terms of ME.

The cubed covariates model had the best predictive ability for each model type, however in the specification with no covariates, the OLS model had the highest overall rank for predictive ability. The OLS model with cubed covariates was one of the best performing models overall (equal ranking with the two-part models with regular or squared covariates). Overall, the OLS and two-part models were the best performing models.

Performance of the mapping algorithm at a range of EPDS scores and thresholds related to postnatal depression is summarised in Table 4. For EPDS scores less than 10 the OLS model with no covariates predicted utility values closer to the observed value than the two-part model with all covariates. In contrast, when the sample was dichotomised at an EPDS score of 10 (nominally representing those with and without depression), the two-part model predicted a mean utility value closer to the observed value than the OLS model. When a threshold of 13 on the EPDS is used to indicate probable depression, the OLS model predicts mean utility values closer to the observed mean than the two-part model. Although when n is large (i.e. EPDS score < 10 or < 13) both models predict mean values close to the observed means. For all examples the standard deviations are higher in the observed data than the predicted values.

**Table 4:** Observed and predicted values at total EPDS scores indicative of depression and depression thresholds

EPDS score	n	Observed utility value	Predicted utility value OLS1	Predicted utility value TPM4
0	107	0.957	0.971	0.934
4	93	0.915	0.912	0.904
8	51	0.854	0.853	0.867
10	42	0.814	0.824	0.847
EPDS threshold				
< 10	869	0.918 (0.13)	0.916 (0.04)	0.918 (0.03)
≥ 10	199	0.765 (0.19)	0.776 (0.05)	0.767 (0.05)
< 13	978	0.904 (0.14)	0.904 (0.05)	0.905 (0.05)
≥ 13	90	0.731 (0.18)	0.733 (0.04)	0.720 (0.03)

OLS1 = ordinary least squares model with no additional covariates; TPM4 = two-part model with cubed covariates

### Validation

The predictive ability of specifications 1 (no covariates) and 4 (cubed covariates) for the OLS and two-part regression models were explored in the three external datasets (described in the “Methods” section).

The characteristics of the external samples are shown in Table 5. For the Minding the Baby study participant level data on age were not available. The mean EPDS score is notably higher in the RESPOND sample who all had a diagnosis of postnatal depression. Across the three samples the one with the lowest mean EPDS score also had the highest mean utility value (SHIP) and the one with the highest mean EPDS score had the lowest mean utility value (RESPOND). The RESPOND study had a very small proportion of women who had full health which may have an impact on the performance of the two-part model which incorporates the probability of having full health in its predictions. There was a negligible proportion of women who had utility values below one, ranging from 0.1% to just below 3%.

**Table 5:** Characteristics of external validation samples

	SHIP	Minding the Baby	RESPOND
	Mean (SD) [min, max]		
Number of observations	1059	319	245
Age at baseline (years)	28.4 (5.3) [18, 43]	Not available^a^	29.4 (6.3) [18, 44]
EPDS score (0–30)	7.3 (2.6) [1,2 2]	8.3 (5.6) [0, 26]	17.5 (3.4) [13, 29]
EQ-5D-3L utility index value	0.914 (0.133) [ – 0.594, 1]	0.821 (0.255) [ – 0.594, 1]	0.687 (0.234) [ – 0.429, 1]
	*N (%)*		
Full health	667 (63.0)	147 (46.1)	7 (2.9)
Negative utility index value	1 (0.1)	9 (2.8)	7 (2.9)

EPDS = Edinburgh Postnatal Depression Scale

^a^An age of 19 years was assumed for all participants (see “Methods” for details)

**Fig. 3:**
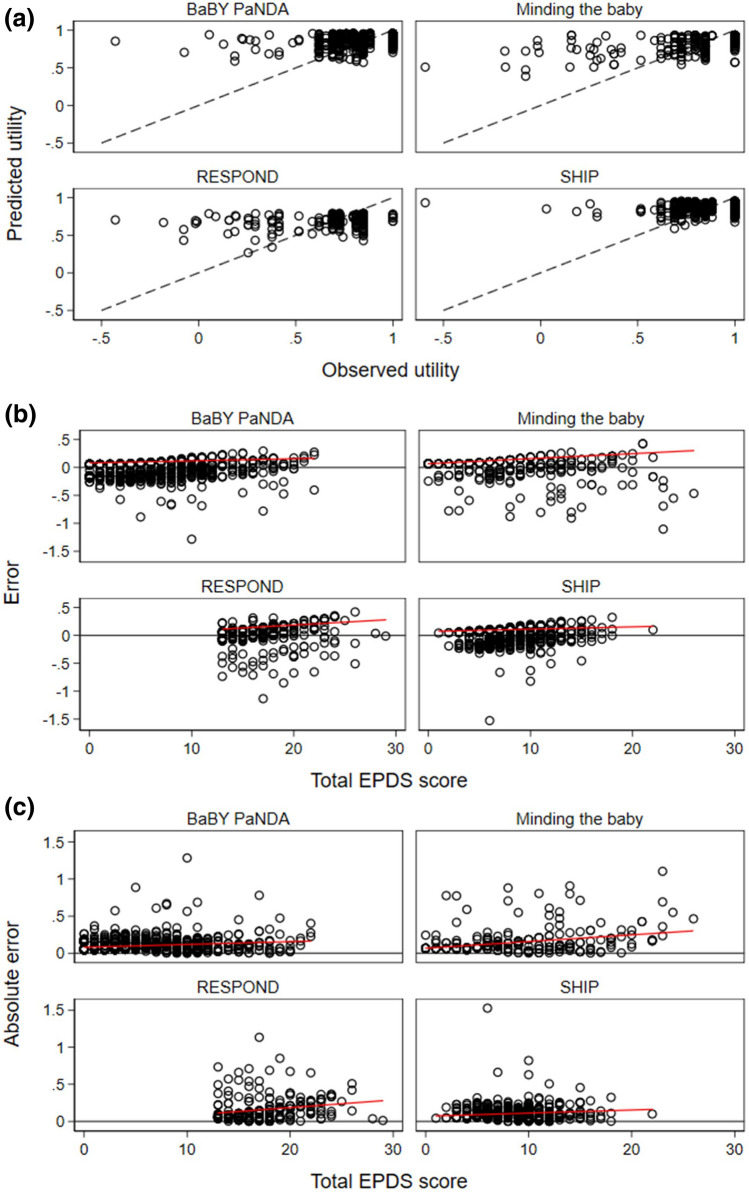
Predicted and observed EQ-5D-3L utility index values (**a**), error (**b**), and absolute error (**c**) for best-performing model (two-part model with cubed covariates)

The observed and predicted values for the best-performing model in the BaBY PaNDA sample (two-part model with cubed covariates) are shown for the estimation sample and validation samples in Fig. 3a. As shown in Fig. 3a, the models perform better in all samples for observed values above 0.5. In the SHIP sample, there are few observations below 0.5; these do not appear to be well predicted by the model (i.e. do not follow the dashed line). In the Minding the Baby sample, again model performance appears better at higher utility values. In this sample there are more observed utility values below 0.5 than in the BaBY PaNDA and SHIP samples and they appear to better follow the perfect prediction (dashed) line. The RESPOND sample (women with postnatal depression) has more observed utility values below 0.5 than the other samples, and between 0.5 and 0.25 the values in this sample are better predicted than in the other samples.

In Fig. 3b and c, the errors and absolute errors in predicted utility values are plotted against total EPDS score for the preferred model. This gives an indication whether the model performs better at particular EPDS scores. In all of the samples, the size of the error increases slightly as EPDS scores increase. In the Minding the Baby and RESPOND samples where there were more EPDS scores above 20, the regression line can be seen to move further from the zero-error line than the other two samples. The same is true for absolute error, with the SHIP sample showing the smallest deviation from the zero-error line.

Quantitative model performance in the estimation and validation samples is summarised in Table 6. For the SHIP sample, the mean predicted utility was lower for all of the models than the observed data, with notably smaller SD than the observed values. For the Minding the Baby sample, the mean predicted utility was higher for all of the models than the observed data, with smaller SDs than the observed values. In the RESPOND sample, the mean predicted utility was higher in 3 models but lower in one model, again with smaller SDs in the predicted than the observed values.

**Table 6:** Model performance and predictive ability for the mapping models in external datasets

STUDY	MODEL	Mean	SD	Min	max	ME	MAE	RMSE	ME rank	MAE rank	RMSE rank	Overall rank
BaBY PaNDA	*Actual data*	*0.890*	*0.150*	* – 0.429*	*1*							
	OLS1	0.890	0.068	0.648	0.971	**0.00000**	0.10253	0.13429	2	1	4	**7**
	TWOPART1	0.890	0.067	0.683	0.963	**0.00000**	0.10254	0.13417	4	2	3	9
	OLS4	0.890	0.069	0.651	0.994	**0.00000**	0.10313	0.13385	3	3	1	**7**
	TWOPART4	0.890	0.069	0.637	0.971	**0.00000**	0.10314	0.13387	1	4	2	**7**
SHIP	*Actual data*	*0.914*	*0.133*	* – 0.594*	*1*							
	OLS1	0.863	0.039	0.648	0.956	0.05048	0.11428	0.13507	4	4	4	12
	TWOPART1	0.883	0.051	0.638	0.973	0.03084	0.10513	0.12872	2	2	2	6
	OLS4	0.867	0.045	0.660	0.949	0.04641	0.11189	0.13323	3	3	3	9
	TWOPART4	0.895	0.051	0.589	0.957	0.01835	0.10078	0.12610	1	1	1	**3**
Minding the Baby	*Actual data*	*0.821*	*0.255*	* – 0.594*	*1*							
	OLS1	0.849	0.083	0.589	0.971	* – *0.02774	0.14178	0.22239	3	2	4	9
	TWOPART1	0.859	0.097	0.597	0.979	* – *0.03810	0.13742	0.22158	4	1	2	7
	OLS4	0.826	0.084	0.633	0.929	* – *0.00482	0.14920	0.22200	1	4	3	8
	TWOPART4	0.836	0.108	0.388	0.936	* – *0.01444	0.14250	0.21316	2	3	1	**6**
RESPOND	*Actual data*	*0.687*	*0.234*	* – 0.429*	*1*							
	OLS1	0.714	0.050	0.545	0.780	* – *0.02769	0.15339	0.22981	4	1	2	7
	TWOPART1	0.698	0.046	0.569	0.767	* – *0.01176	0.15651	0.22894	2	2	1	**5**
	OLS4	0.706	0.038	0.635	0.786	* – *0.01981	0.15672	0.23206	3	3	4	10
	TWOPART4	0.685	0.085	0.267	0.793	0.00180	0.15943	0.23031	1	4	3	8

The assessment of model performance is based on ME, MAE, RMSE ranks, with ‘1’ indicating the closest fit to the observed data. SD = standard deviation; ME = mean error; MAE = mean absolute error; RMSE = root mean squared error.

Model specification: 1 = no covariates; 4 = cubed covariates. Covariates = age, total EPDS score

Bold numbers indicate a mean error of 0 to at least 5 decimal places

The best-performing model in the two general population validation samples (SHIP and Minding the Baby) was the two-part model with cubed covariates. It should be noted that in the Minding the Baby sample there was little difference between the models which reflects that the same age was assumed for all participants. In contrast in the RESPOND sample, of women with postnatal depression, the two-part model with no additional covariates was the best-performing model.

The MAEs were similar for the BaBY PaNDA sample and the SHIP sample, although were slightly higher (by around 0.04) in the Minding The Baby sample and highest in the RESPOND sample (by around 0.05). This was also the case with the RMSEs, with similar values for SHIP and BaBY PaNDA, and higher values for the Minding The Baby (by around 0.09) and RESPOND (by around 0.1) samples. For the SHIP sample the MAEs equate to a percentage error of approximately 6% of the overall utility index value range and the RMSEs of approximately 8%. For the Minding the Baby sample the MAEs equate to a percentage error of approximately 9% of the overall utility index value range and the RMSEs of approximately 13%. For the RESPOND sample the MAEs equate to a percentage error of approximately 11% of the overall utility index value range and the RMSEs of approximately 16%.

Overall, the models perform best in the SHIP sample and least well in the RESPOND sample who all had postnatal depression. As shown in Fig. 3, the preferred model performs reasonably well at observed utility values above 0.5, worse at values between 0.5 and 0, and poorly at values below 0. As shown in Table 4, the proportion of women in these samples with utility values below 0 are between 0.1% and 2.9%. The MAE and RMSE of the best performing model were lower in the SHIP sample than the estimation sample which suggests good external validity in some populations at least. The model performs better when utility values are above 0.5 therefore may perform less well in samples where all participants have postnatal depression.

### Overall recommendation

The two-part model performs well when predicting utility values from total EPDS score. Inclusion of covariates (age, age^2^, age^3^, EPDS^2^, and EPDS^3^) improve the predictive ability of the model. The model performs better in samples where participants are in health is good (high utility values) and depression symptoms are not severe (EPDS score < 20).

The mapping algorithms with and without covariates for the best-performing model and the OLS model can be implemented via Excel and these are provided in Supplementary Material.

## Discussion

This paper reports the development and validation of a mapping algorithm between the EPDS and 3-level version of the EQ-5D (EQ-5D-3L). To our knowledge this has not been done before [23]. The ME and RMSE indicated that the two-part model with cubed covariates (EPDS score and age) performed best in predicting health utility index values in perinatal women (ranking 1st and 2nd on these measures, respectively). This model type also performed well in 3 data samples which were external to the estimation sample. With the interactive and freely accessible spreadsheet produced (available in Supplementary Material), health utility values can be estimated directly from the EPDS when primary EQ-5D data have not been collected. There was a moderate correlation between EPDS score and utility score and weak correlations between EPDS score and individual EQ-5D domain responses (other than anxiety which was moderately correlated). This meant that mapping between the EPDS and the individual domains of the EQ-5D was not appropriate. Higher utility values were more reliably mapped than lower values. The minimum predicted values from all the models were above 0 (whereas observed utility values did include negative values).

Our algorithm can be used by researchers who have not collected EQ-5D data but want to be able to report cost-effectiveness in terms of preference-based health utility according to policy-makers' guidance (e.g. NICE [16]). Although we acknowledge that deriving health utility directly from the EQ-5D is generally considered preferable where possible. We envisage that health economists building state transition models relevant to perinatal mental health may wish to estimate utility values for "depressed" and "not depressed" health states. One way of defining these health states is through the application of EPDS threshold scores. In this paper we demonstrated that the best-performing models predict mean utility values which are similar to the observed data around the commonly used threshold scores. If researchers only had access to EPDS data for a sample and wanted to calculate utility for depression health states, then they could use our interactive spreadsheet to do this for their specific sample.

External validation of the mapping algorithm is a key strength of this work. In one external validation sample (the RESPOND study) all of the participants had a diagnosis of postnatal depression and the mean observed utility value was notably lower than the other samples. In this sample the two-part model with no covariates was the best-performing model. This suggests that when there is less variation in EPDS scores (i.e. everyone had high scores), age may have less influence on health utility than in other samples. The MAE and RMSE of the predicted utility values for this sample were higher than the other samples. For all the samples, predicted utility values were closer to observed values at higher utility values. Together this suggests that the algorithms would perform better in samples with higher utility on average. This is potentially less of an issue in the context of the perinatal period than it may be in other contexts (e.g. samples of older people or people with multiple long-term health conditions). Age is a key determinant of health utility [37] and there is relatively limited variation in age in this context. In England for example, the mean age of women giving birth is around 30 [3] with mean general population utility values for people of this age being between 0.938 (95% CI 0.935–0.941) (adults under 30) and 0.915 (95% CI 0.907–0.921) (people aged 30–35) [37].

There are some limitations of our mapping algorithm which should be considered when interpreting the findings. The value set used to derive utility index values from EQ-5D-3L responses in the estimation and validation samples was based on data from the United Kingdom (UK) collected in the 1990s [27]. It is unknown how well the health state valuations elicited 20 + years ago represent current population preferences. Similarly, the algorithm may not be fully generalisable to value sets from other countries. Another consideration is that the newer 5-level version of the EQ-5D (EQ-5D-5L) which asks respondents to rate their health over 5 (rather than 3) levels is increasingly being used to estimate health utility [17]. Further work to map between the EPDS and utility values derived from the 5-level version of the EQ-5D would be a useful addition to our algorithm. In the context of mental health, the methods used can have an important impact on utility values derived from the EQ-5D-5L [38] and so this would also be an important consideration in future mapping work.

Including age as a covariate in the algorithm produced largely similar results to when it was not included. This is possibly due to the limited age range of participants in the sample (i.e. women of child-bearing age). This means that the algorithm can still be used when no additional covariates are available making it widely applicable e.g. in meta-analyses. However, EPDS score, and age do not perfectly predict utility values which means that unmeasured characteristics may play an important role in the relationship between EPDS score and utility values. For example, deprivation is associated with health utility [39] and family income is associated with a higher risk of developing PND [8]. Similarly, a traumatic birth experience is associated with an increased risk of PND [40] and if there are lasting physical impairments following a difficult birth then someone may have problems on multiple EQ-5D domains (e.g. mobility, usual activities) in addition to the anxiety/depression domain. Future exploration of the impact of unmeasured factors on the performance of the mapping algorithm may enable more precise prediction of utility values from the EPDS, however this must be balanced against the risk of over-fitting the algorithm to an estimation sample and ending up with a complex algorithm that can only be used by researchers with similarly complex datasets.

### Supplementary Information

Below is the link to the electronic supplementary material.

Supplementary file1 (XLSX 27 KB)

## Data Availability

The data that support the findings of this study are available upon reasonable request from the original researchers who conducted the primary research studies (BaBY PaNDA [Gilbody]; RESPOND [Sharp]; Minding The Baby [Fearon]).
